# Inhibition of the NLRP3 Inflammasome Activation by Manoalide Ameliorates Experimental Autoimmune Encephalomyelitis Pathogenesis

**DOI:** 10.3389/fcell.2022.822236

**Published:** 2022-02-16

**Authors:** Cong Li, Hualong Lin, Hongbin He, Ming Ma, Wei Jiang, Rongbin Zhou

**Affiliations:** ^1^ Department of Geriatrics, First Affiliated Hospital of USTC, Division of Life Sciences and Medicine, CAS Key Laboratory of Innate Immunity and Chronic Disease, School of Basic Medical Sciences, University of Science and Technology of China, Hefei, China; ^2^ CAS Centre for Excellence in Cell and Molecular Biology, University of Science and Technology of China, Hefei, China

**Keywords:** NLRP3 inflammasome, manoalide, inhibitor, inflammatory diseases, EAE

## Abstract

The activation of NLRP3 inflammasome leads to cell pyroptosis and inflammatory cytokines secretion and gets involved in the development of many diseases, such as neuroinflammation and metabolic syndrome, but the drugs targeting NLRP3 are not clinically available for now. Through screening the small molecule library, we found that manoalide is a highly selective small molecule inhibitor of NLRP3. Mechanismly, manoalide inhibited the NLRP3 inflammasome activation by acting downstream of potassium efflux, chloride efflux and mitochondrial dysfunction. Moreover, manoalide blocked the interaction between NEK7 and NLRP3 by covalently binding to Lys 377 of the NLRP3 protein. Treatment of manoalide relieved the pathogenesis of experimental autoimmune encephalomyelitis (EAE) in mice. Thus, our results identify manoalide as a selective and covalent NLRP3 inhibitor and suggest it has the potential for the treatment of NLRP3-associated diseases.

## Introduction

The NLR family pyrin domain containing 3 (NLRP3) inflammasome plays an important role in innate immune response by forming an intracellular protein complex consisted of NLRP3, ASC and pro-caspase-1 (Chen et al., 2009; Davis et al., 2011). Induced by components of pathogens or danger signals released in tissue damage, the activation of NLRP3 inflammasome initiates cell pyroptosis and the secretion of mature IL-1β and IL-18, leading to inflammatory response (Martinon et al., 2009; Dick et al., 2016). Although the release of pro-inflammatory cytokines provides protection to the host during infections, the exceeding activation of inflammasome will further induce tissue damage. Recently, it has been found that the aberrant activation of NLRP3 inflammasome is associated with a variety of diseases, such as metabolic syndrome, neuroinflammation, and inflammatory bowel disease (IBD) (Sharma and Kanneganti 2021). Therefore, development of the potential inhibitors of NLRP3 inflammasome attracts tremendous attention. Strategies for inhibition of NLRP3 inflammasome contains inhibition of the NLRP3 expression, IL-1β, caspase1 and NLRP3 activaiton (Man et al., 2015). Limiting TLR-mediated and TNF-mediated priming signal reduces NLRP3 expression, but inhibits activation of NF-κB at the same time (Bahia et al., 2015). Anakinra, canakinumab and rilonacept are approved for therapeutic use to inhibit IL-1, which is one of the main cytokine released during inflammasome activation and the effector molecule in NLRP3-associated diseases (Dinarello et al., 2012). However, inhibition of IL-1 has no effects on pyroptosis and other pro-inflammatory factors (IL-18, HMGB1) released during the inflammasome activation. The activation of NLRP3 inflammasome is not the only source of IL-1β *in vivo*, so blocking the IL-1 signal increases risk of infection. Inhibition of caspase1 could effectively abrogate the formation of inflammasome complex (Wannamaker et al., 2007; Boxer et al., 2010). However, caspase1 is an effector molecule in many kinds of inflammasomes. Caspase1 inhibition may also increase the risk of infection. Instead of molecules targeting products or other components of the inflammasome, inhibitors directly targeting the NLRP3 protein possess less immunosuppressive and off-target effects. Several molecules targeting NLRP3 have showed inhibitory effects on the activation of inflammasome through *in vitro* and *in vivo* studies, including MCC950, OLT1177, CY-09, Oridonin, and RRx-001 (Coll et al., 2015; Jiang et al., 2017; He et al., 2018; Marchetti et al., 2018a; Chen et al., 2021). CY-09 and oridonin have not undergone clinical trials yet. OLT1177 is in phase II clinical trial and RRx-001 is in phase III clinical trial (Marchetti et al., 2018b; Cabrales 2019). Thus, the inhibitor targeting NLRP3 is not clinically available for now.

Manoalide, first isolated from the sponge *Luffariella variabilis* in the early 1980’s, is a marine natureal sesterterpene and has potent antibiotic, analgesic and anti-inflammatory effects (De Silva and Scheuer 1980; Wang et al., 2019). Manoalide could permanently block phospholipase A2 (PLA2), phospholipase C (PLC) and NS3(Bennett et al., 1987b; Folmer et al., 2010; Salam et al., 2012). Previously, it has been demonstrated that manoalide represses the inflammation by irreversible interaction with lysine residues on PLA2. The binding is achieved through two aldehyde functional groups of manoalide, including γ-hydroxybutenolide and α-hydroxydihydropyran. The γ-hydroxybutenolide ring promotes the reaction between manoalide and PLA2, and the hemiacetal in α-hydroxydihydropyran is needed for permanent binding (Glaser et al., 1989). However, the IC50 value of manoalide inhibition of PLA2 in the mouse ear homogenates is 60 μM, which is 2.5-fold greater than the ED50 required to prevent phorbol ester-induced inflammation (Bennett et al., 1987a), indicating that manoalide might inhibit the inflammation through other targets.

In our study, we reported that manoalide specifically inhibits the NLRP3 inflammasome activation by blocking the NEK7-NLRP3 interaction. Manoalide interacted with NLRP3 covalently and targeted lysine 377 of the NLRP3 NACHT domain. In mouse EAE model, manoalide treatment attenuated the inflammation in CNS and had great therapeutic effects. Our study identified manoalide as a new potential therapeutic agent for NLRP3-related diseases.

## Materials and Methods

### Mice

Specific pathogen-free (SPF) C57BL/6J mice were purchased from the Shanghai SLAC Laboratory Animal Ltd. Corp. (Shanghai, China). Nlrp3^−/−^ mice were described previously (Martinon et al., 2006). All mice were housed under a 12-h light/12-h dark cycle (light on at 8:00 and off at 20:00). Unless otherwise specified, all experiments used male mice between 8 and 12 weeks old. All animal experimental protocols were reviewed and approved by the Animal Care Committee of the University of Science and Technology of China.

### Reagents and Antibodies

Manoalide (sc-200733) was from Santa Cruz. Nigericin, MSU, ATP, and Poly (dA:dT) were from Sigma-Aldrich. Pam3CSK4, ultrapure LPS, MitoTracker, MitoSOX, MQAE, DAPI, Lipofectamine RNAiMAX and Lipofextamine 2000 were from Invitrogen. Mitotracker green and Mitotracker deep red were from Thermo Fisher Scientific. CY-09 was synthesized by Dr. Xianming Deng (Xiamen University, Xiamen, China) (Jiang et al., 2017). C3 Toxin (*Clostridium botulinum* ADP-ribosylating C3 toxin) was a gift from Tengchuan Jin (University of Science and Technology of China, Hefei, China) (Xu et al., 2014). The *Salmonella* strain (*Salmonella typhimurium*) was a gift from Dr. Cai Zhang (Shandong University, Shandong, China). Protein G Agarose (16-266) was from Millipore. Anti-Flag beads (A2220), anti-Flag antibody (F2555) and anti-VSV antibody (V4888) were from Sigma-Aldrich. Anti-mouse NLRP3 antibody (AG-20B-0014) and anti-mouse caspase-1 antibody (AG-20B-0042) was purchased from AdipoGen. Anti-mouse IL-1β antibody (AF-401-NA) was from R&D Systems. Anti-mouse NEK7 antibody (ab133514) was from Abcam. Anti-mouse ASC antibody (67824S) was from Cell Signaling Technology. Anti-β-actin antibody (66009-1-Ig) was from Proteintech Group. Phospho-p44/42 MAPK (Erk1/2) (Thr202/Tyr204) (E10) Mouse mAb (#9106), Phospho-SAPK/JNK (Thr183/Tyr185) Antibody (#9251), Phospho-p38 MAPK (Thr180/Tyr182) Antibody (#9211) and Phospho-IκBα (Ser32/36) (5A5) Mouse mAb (#9246) were from Cell Signaling Technology.

### Cell Culture and Stimulation

BMDMs were isolated from 6-8 weeks-old mice bone marrow and cultured in 37 C for 4-5 days in DMEM containing 10% FBS (Biological Industries), 1 mM sodium pyruvate, 2 mM l-glutamine in the presence of 20 ng/ml recombinant M-CSF (Novoprotein). HEK-293T were from American Type Culture Collection (ATCC), cultured in DMEM with 1% penicillin-streptomycin solution and 10% FBS. PBMCs from human peripheral blood were isolated by Human Lymphocyte Separation Medium (Solarbio). PBMCs and THP-1 were cultured in RPMI 1640 medium with 1% antibiotics and 10% FBS.

5 × 10^5^ cells/mL BMDMs or 1 × 10^6^ cells/mL PBMCs were seeded in a 12-well plate and cultured overnight. The cells were primed with 50 ng/ml LPS (for canonical inflammasome activation) or 400 ng/ml Pam3CSK4 (for non-canonical inflammasome activation) for 3 h. Then, the cells were treated with manoalide for 30 min. To induce the NLRP3 inflammasome activation, the cells were stimulated with nigericin (3 μM) for 20 min, ATP (2.5 mM) for 30 min, or MSU (150 μg/ml) for 3 h. To induced the non-canonical inflammasome activation, the cells were primed with 400 ng/ml Pam3CSK4 for 3 h, transfected with LPS (500 ng/ml, using lipo 2000) and cultured for 16 h. To induce the activation of the AIM2 inflammasome, BMDMs were transfected with Poly (dA:dT) for 4 h. To induce the activation of the NLRC4 inflammasome, the cells were infected with *S. typhimurium* (multiplicity of infection (MOI) = 10) for 4 h. To induce the Pyrin inflammasome activation, the cells were stimulated with C3 toxin (0.5 μg/ml) for 6 h. Cell supernatants were detected by ELISA, at the same time, the supernatants and cell lysates were collected for immunoblot analysis.

THP-1 were seeded in a 12-well plate with 100 nM PMA and cultured overnight. The cells were primed with 50 ng/ml LPS for 3 h, Then, the cells were treated with manoalide for 30 min. To induce the NLRP3 inflammasome activation, the cells were stimulated with nigericin (3 μM) for 20 min.

### Bacteria Culture

*S. typhimurium* was seeded in trypticase soy broth (TSB) medium and cultured in 37 C with 200 rpm overnight. The suspensions were diluted by multiples and coated on solid TSB medium to measure the density of bacteria.

### ELISA

Supernatants of PBMCs were assayed for human IL-1β (BD, 557953) and human TNF-α (R&D, DY-210) according to the manufacturer’s instructions. Supernatants from BMDMs were assayed for mouse IL-1β (R&D, DY-401), mouse TNF-α (R&D, DY-410) and mouse IL-6 (R&D, DY406) according to the manufacturer’s instructions.

### Measurement of Intracellular Potassium and Chloride Concentrations

To measure the concentration of intracellular potassium, BMDMs were seeded in 6-well plates overnight and stimulated to induce the NLRP3 inflammasome activation in the presence or absence of manoalide pretreatment. Then, remove the medium and wash the cells with pre-chilled K^+^ PBS. After that, the cells were lysed by ultrapure HNO_3_. The lysates were boiled for 30 min to obtain the precipitate and then dissolved in ddH_2_O. The potassium concentration was measured by inductively coupled plasma emission spectrometry with PerkinElmer Optima 7300 DV spectrometer.

To measure the concentration of intracellular chloride, BMDMs were seeded in 12-well plates overnight and stimulated to induce the NLRP3 inflammasome activation in the presence or absence of manoalide pretreatment. Then, remove the medium and lyse the cells with ddH2O at 37 C for 15 min. The lysates were stored at −80 °C for 30 min and centrifuged at 8,000 rpm for 5 min after thawing. Transfer the supernatants to a new 1.5  ml EP tubes. After that, 40 μL MQAE (10 μM) was mixed with 40 μL supernatant and the absorbance of the mixed solution were measured by a BioTek Multi-Mode Microplate Reader (Synergy2). Calculating the corresponding chloride concentration based on the fluorescence value of each sample (Tang et al., 2017).

### NLRP3 ATPase Activity Assay

Purified recombinant human NLRP3 proteins (1.4 ng/uL) were incubated with manoalide in reaction buffer at 37°C for 15 min (Jiang et al., 2017). Add Ultra-Pure ATP (25 μM) to the mixture and incubate at 37 C for 40 min. Add ADP-Glo reagent and incubate at room temperature for 40 min to terminate kinase reaction and clear unreacted ATP. Then, add Kinase Detection Reagent into the mixture and incubate at room temperature for 30 min. The amount of ATP converted into adenosine diphosphate (ADP) was detected by the luminescent ADP detection. The results were expressed as the percentage of residual enzyme activity and carrier treatment enzyme (Jiang et al., 2017).

### Confocal Microscopy

BMDMs (2 × 10^5^/ml) were seeded on coverslips in 12-well plates overnight. BMDMs were treated with manoalide and stimulated to induce the activation of NLRP3 inflammasomes by nigericin. Stain the cells with MitoSOX (5 μM) or MitoTracker (50 nM). Then, remove the supernatants and wash the cells with chilled PBS for three times. Fix the cells with 4% PFA at room temperature for 20 min and wash with PBST for three times. Use DAPI to stain the nuleus. Confocal microscopic analyses were performed by Zeiss LSM 700.

### FACS Analysis

THP-1 (1 × 10^6^/ml) were seeded in 6-well plates overnight. BMDMs were treated with manoalide and stimulated to induce the activation of NLRP3 inflammasomes by nigericin. Stain the cells with MitoSOX (50 μM) or Mitotracker green (50 nM) and Mitotracker deep red (50 nM) for 30min. Cells were washed with PBS solution and re-suspended in PBS solution for FACS analysis (Zhou et al., 2011).

### Gene Knockdown in BMDMs With siRNA

Gene knockdown in BMDMs has been described previously (Chen et al., 2021). The siRNA sequences were as follows:

siPla2g4a: 5′-GCA​CAG​CTA​CAT​TCC​CTG​TAT-3’;
siPla2g7: 5′-CTT​GGC​ATC​TAA​TGG​GTT​TAT-3’;
siPla2g15: 5′-CAC​GCC​AAA​CTC​TTT​CTA​CTA-3’;
siPlcg2: 5′-GCA​AAG​GCA​TAT​TGG​ATC​TTA-3’.

#### Quantitative Real-Time PCR

Total RNA of BMDMs and tissues were extracted by TsingZol Total RNA extraction reagent (Tsingke). 800 ng RNA per sample was reverse transcribed by Thermo Scientific RevertAid MM (Thermo Fisher SCIENTIFIC). Quantitative PCR was performed in Roche LightCycler 96 by using 2X Universal SYBR Green Fast qPCR Mix (Abclonal). GAPDH was used as a reference gene. The sequences of the gene-specific primers were as follows:

Mouse Pla2g4a forward, TCC​TTA​TCA​GCA​CAT​TAT​AGT​GGA; Mouse Pla2g4a reverse, GTC​TCA​TTC​CAC​ACG​GGG​TT; Mouse Pla2g7 forward, AAA​CTG​CAG​GCG​CTT​TTC​TG; Mouse Pla2g7 reverse, CGA​CGG​GGT​ACG​ATC​CAT​TT; Mouse Pla2g15 forward, TCT​ACT​AAT​GAT​GCT​GGC​AGA​CCT; Mouse Pla2g15 reverse, TAG​GGC​CCG​TTT​TCA​TTT​GG; Mouse Plcg2 forward, CCC​AGA​TGG​TGG​CAC​TCA​AT; Mouse Plcg2 reverse, CCA​GGG​GCA​TCG​GAT​CAT​AC; Mouse IL1b forward, TGC​CAC​CTT​TTG​ACA​GTG​ATG; Mouse IL1b reverse, AAG​GTC​CAC​GGG​AAA​GAC​AC; Mouse IL-6 forward, GTC​CTT​CCT​ACC​CCA​ATT​TCC; Mouse IL-6 reverse, GCA​CTA​GGT​TTG​CCG​AGT​AGA; Mouse *Tnf-α* forward, CGA​TGG​GTT​GTA​CCT​TGT​C; Mouse *Tnf-α* reverse, CGG​ACT​CCG​CAA​AGT​CTA​AG; Mouse Gapdh forward, GGT​GAA​GGT​CGG​TGT​GAA​CG; Mouse Gapdh reverse, CTC​GCT​CCT​GGA​AGA​TGG​TG.

### ASC Oligomerization Detection

BMDMs were plated in 6-well plates and stimulated as described previously. The supernatants were collected for western blot analysis and the cells were lysed with TBS buffer (50 mM Tris-HCl, 150 mM NaCl, 0.5% Triton X-100, pH 7.4) on a shaker for 30 min in 4 C. Then, lysates were centrifuged at 6,000 × g/4 C for 15 min and the supernatants were removed. The pellets were washed twice with TBS buffer and resuspended in 500 uL TBS buffer. The resuspended pellets were incubated with 2 mM disuccinimidyl suberate (DSS, Thermo Fisher Scientific) for cross-linking at 37 C for 30 min and the pellets were turned over every 10 min. After that, the samples were centrifuged at 6,000 × g/4 C for 15 min. The crosslinked pellets were dissolved in 30 uL sample buffer, boiled at 100 C for 15 min, and analyzed by immunoblotting.

### Plasmid Constructions

PCR reaction was performed by using PrimeSTAR^®^ Max DNA polymerase (TaKaRa). Gene shear was performed by restriction enzymes (Thermo Fisher Scientific). And gene recombination was performed using the ClonExpress^®^ II One Step Cloning Kit (Vazyme). To the NACHT mutant, primers were used to get pCR3-Flag-NLRP3 linear DNA, and the recombinant plasmid was generated by using ClonExpress^®^ II one-step cloning kit (Vazyme). To construct pLEX-NLRP3(C405A), primers were used to get pLEX-NLRP3 linear DNA. and the recombinant plasmid was generated by using ClonExpress^®^ II one-step cloning kit (Vazyme).

### Co-Immunoprecipitation Assay

For exogenous co-immunoprecipitation, the HEK-293T cells were seeded in 6-well plates and cultured overnight. Then, the cells were transfected with plasmids via polyethylenimine and cultured for 24 h. Lyse the cells with 300 uL NP-40. Incubate the lysates with anti-Flag antibody-coated beads and analyze by Western blotting.

For endogenous co-immunoprecipitation, BMDMs were seeded in 6-well plates and cultured overnight. Stimulate the cells with nigericin as described previously. Then, lyse the cells with 300 uL NP-40. Incubate the lysates with Protein G Mag Sepharose overnight at 4 C. Incubate the lysates with primary antibodies or IgG as control. The proteins bound by primary antibodies were precipitated by protein G beads and then assessed by Western blotting.

### Protein Expression and Purification

The HEK-293T cells were transfected with His-GFP-NLRP3 plasmid and cultured for 48 h. The cells were lysed in lysis buffer (50 mM Hepes (pH 7.4), 150 mM NaCl, and 0.4% CHAPS) for 30 min. After sonication, the lysates were centrifuged at 14,000 rpm for 15 min at 4 C to remove the insoluble fraction. The His-GFP-NLRP3 protein was incubated with nickel-nitrilotriacetic acid matrices (QIAGEN) at room temperature for 45 min. Histidine pull-down products were washed twice with lysis buffer and eluted by eluted buffer. The eluted products were filtered through a 0.45-µm syringe filter and concentrated by the ultrafiltration device (Merck Millipore) to remove proteins <10 kD. Then, 5 ml of the final eluted products were injected into a Superdex 200 10/300 GLcolumn (GE Healthcare). The His-GFP-NLRP3 protein was further purified in 50 mM Hepes (pH 7.4), and 150 mM NaCl on an AKTA purifier (GE Healthcare). Each tube of fraction (10 µL each) was resolved by SDS-PAGE gels and detected by anti-His antibody. Solution in the tubes containing His-GFP-NLRP3 protein was collected together and subjected to concentration by ultrafiltration device (UFC910024; Merck Millipore) to remove proteins <100 kD.

### Microscale Thermophoresis

The three-fold serially diluted manoalide (from 0.56 to 3,703.7 nM) was incubated with 200 nM purified His-GFP-NLRP3 protein in assay buffer (50 mM Hepes, 10 mM MgCl_2_, 100 mM NaCl (pH 7.5) and 0.05% Tween 20) at room temperature for 30 min. Then, the samples were loaded into the NanoTemper glass capillaries for MST analysis under 80% MST power and 100% LED power. The K_D_ values were calculated by Monolith NT.115 instrument (NanoTemper Technologies) and NanoTemper software.

### Drug Affinity Responsive Target Stability Assay

DARTS was performed as described previously (Coll et al., 2019). BMDMs were seeded at 10-cm dishes and cultured overnight. Then, the cells were treated with LPS (50 ng/ml) for 3 h. Remove the medium and lyse the cells with NP-40 lysis buffer. HEK-293T were transfected with plasmid and cultured for 24 h. Then the cells were lysed with NP-40 lysis buffer. The lysates were centrifuged at 12000 × g at 4 C for 10 min. The concentration of protein was measured by Pierce BCA Protein Assay Kit (Beyotime). The lysates were incubated with manoalide at the indicated concentrations with rotation at 4 C overnight. Add pronase into the lysates (25 ng enzyme for 1 μg protein, Sigma), and incubate at room temperature for 30 min. The 3 × SDS loading buffer was added to stop the reaction and then the samples were analyzed by immunoblotting.

### NLRP3 Reconstitution

The cDNA encoding WT NLRP3 or mutant NLRP3 was subcloned into the lentiviral pLEX vector (Thermo Fisher Scientific). The HEK-293T cells were transfected with empty pLEX vector or pLEX containing the indicated plasmid and the packaging vector. After 24 h, remove the supernatants and add fresh medium. After 24 h, the supernatants were collected. Nlrp3^−/−^ BMDMs were seeded in 12-well plates and cultured overnight. Then, replace the supernatant with the collected virus-containing supernatants and culture the cells for 48 h. After that, BMDMs were stimulated and analyzed as described previously.

### Induction and Assessment of Experimental Autoimmune Encephalomyelitis

10-week-old C57BL/6 male mice were emulsified with MOG_35-55_ peptide (300 μg per mouse) in CFA containing 5 mg/ml of heat-killed *Mycobacterium tuberculosis* (500 μg per mouse) for subcutaneous immunity. Mice were injected with Pertussis toxin (150 ng per mouse) via retro-orbital venous plexus injection on day 0 and day 2. Manoalide had been administered i. p. to mice (5 mg/kg) every 3 experimental autoimmune encephalomyelitisdays since day 0. Control mice were administered vehicle (PBS) at the same time. Observe and record the clinical signs of the mice every day. The severity of the disease was scored as follows: 0, no abnormalities; 1, limp tail or waddling gait with tail tonicity; 2, wobbly gait; 3, hind limb paralysis; 4, hind limb and forelimb paralysis; 5, death. To analyze the infiltration of immune cells in CNS, both the brain and spinal cord were harvested from mice perfused with PBS, and mononuclear cells were isolated by 30% Percoll separation.

### Histopathology

The spinal cords were fixed in 4% PFA overnight and sliced after embedding in paraffin. Sections were stained with hematoxylin & eosin (H&E) and Luxol Fast Blue (LFB). Slides were examined under a Nikon ECL IPSE Ci biological microscope. Images were acquired with a Nikon DS-U3 color digital camera.

### Statistical Analyses

All values were expressed as mean ± SEMs. Statistical analysis was performed by using unpaired *t*-test (Graphpad Software). All data points were not excluded. Researchers were not blinded during sample collection or data analysis. Sample sizes were selected on the basis of preliminary results to ensure an adequate power. When *p*-value < 0.05, the data were considered significant.

## Results

### Manoalide Blocks the NLRP3 Inflammasome Activation

**FIGURE 1:**
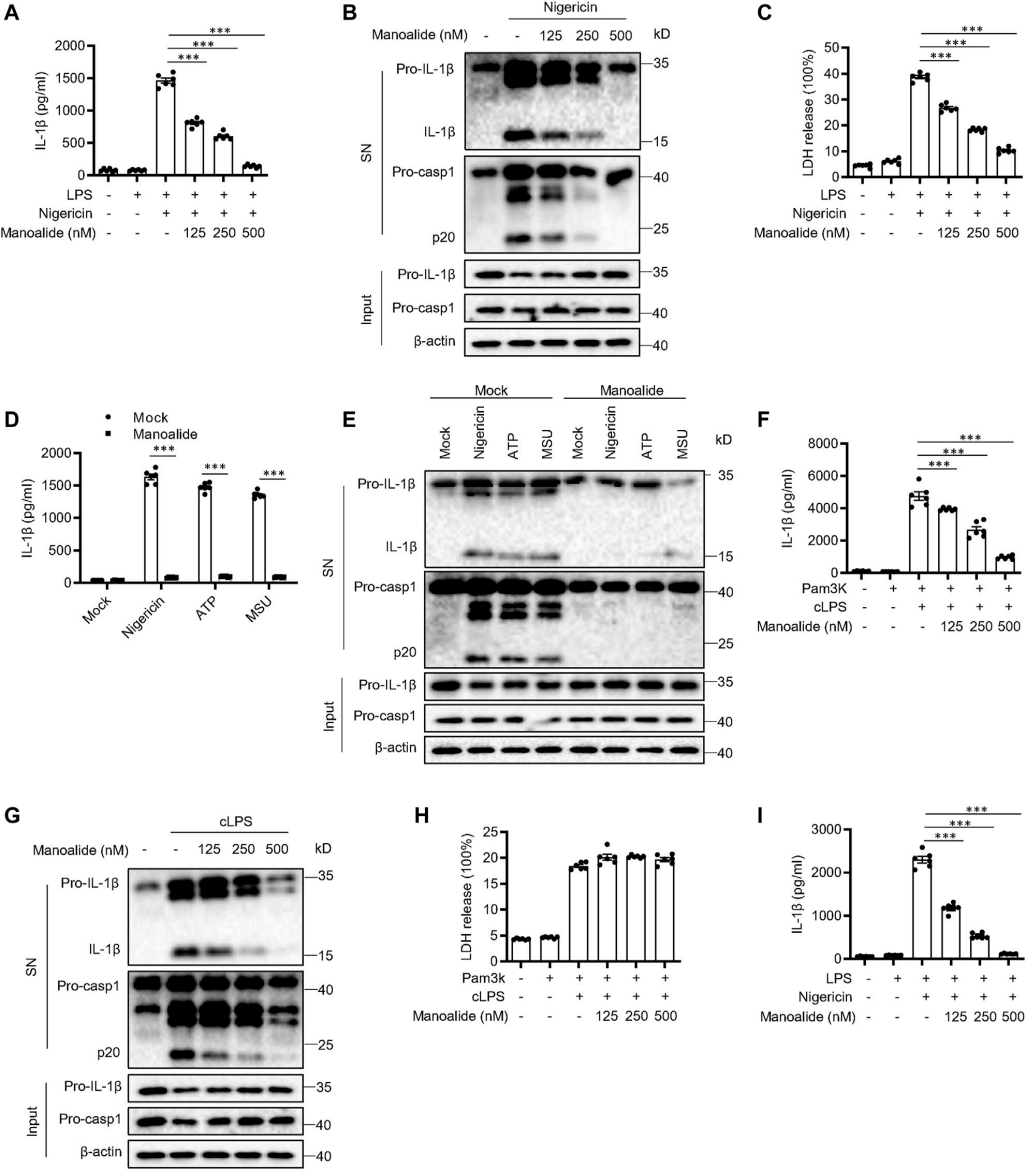
Manoalide inhibits the NLRP3 inflammasome activation. **(A-C)** BMDMs were primed with LPS for 3 h and then were pretreated with 125, 250, 500 nM manoalide before stimulation with nigericin for 15 min **(A)** ELISA of mature IL-1β in supernatant from BMDMs. **(B)** Western blots analysis of supernatant (SN) and cell lysates (Input) from BMDMs. **(C)** LDH release in the supernatant from BMDMs. **(D)** ELISA of mature IL-1β and **(E)** Western blots of SN and Input from BMDMs primed with LPS and stimulated with nigericin, MSU and ATP with or without 500 nM manoalide. **(F-H)** BMDMs were primed with Pam3K for 3 h and then were pretreated with 125, 250, 500 nM manoalide before stimulation with cytosolic LPS (cLPS) for 16 h. **(F)** Production of mature IL-1β in supernatant from BMDMs. **(G)** Western blots analysis of supernatant (SN) and cell lysates (Input) from BMDMs. **(H)** LDH release in the supernatant from BMDMs. **(I)** ELISA of mature human IL-1β in supernatant from PBMCs primed with LPS for 3 h and then were pretreated with 125, 250, 500 nM manoalide before stimulation with nigericin. Data are derived from three independent experiments **(A,C,D,F,H,I)** and displayed as mean and SEM (*n* = 6) or represent three independent experiments **(B,E,G)**. Statistically significant difference was calculated by unpaired Student’s t-test: ^***^ *p* < 0.001.

To test whether manoalide affects the activation of NLRP3 inflammasome, we examined the influence of manoalide on caspase-1 cleavage and IL-1β release in bone marrow-derived macrophages (BMDMs). We treated LPS-primed BMDMs with 125 nM, 250 and 500 nM manoalide for 30 min, and then stimulated the cells with nigericin which is a classic agonist of the NLRP3 inflammasome. Manoalide had a dose-dependent inhibitory effect on the cleavage of caspase-1, secretion of IL-1β/IL-18 and cell pyroptosis (Figures 1A–C and Supplementary Figure S1A). However, manoalide did not affect the secretion of IL-6 and TNF-α induced by LPS (Supplementary Figure S1B,C). Since the NLRP3 inflammasome can be activated by a variety of stimulators, we also explored whether manoalide can inhibit the activation of NLRP3 inflammasome by other agonists. Our data demonstrated that manoalide inhibits the cleavage of caspase-1 and the release of IL-1β induced by ATP and monosodium urate crystals (MSU) (Figures 1D,E). Moreover, manoalide also inhibited the secretion of IL-1β/IL-18 and the cleavage of caspase-1 caused by the activation of noncanonical NLRP3 inflammasomes triggered by cytosolic LPS (cLPS) (Figures 1F,G and Supplementary Figure S1D). But manoalide had no effects on the cell death caused by cLPS (Figure 1H). In addition, We test the effect of manoalide on the activation of noncanonical NLRP3 inflammasome in casp11^−/−^ BMDM. Consistent with previous reports, the activation of noncanonical NLRP3 inflammasome is inhibited in casp11^−/−^ BMDM, and manoalide also had no apparent effect on the process (Supplementary Figures S1E,F). Our results indicated that manoalide is a broad-spectrum, potent inhibitor of the NLRP3 inflammasome. Next, we researched whether manoalide has the same inhibitory effect on human cells. Manoalide inhibited the activation of NLRP3 inflammasome induced by LPS and nigericin in peripheral blood mononuclear cells (PBMCs) (Figure 1I, Supplementary Figure S1G), while it did not affect the production of TNF-α (Supplementary Figures S1H,I). Taken together, we found that manoalide can inhibit NLRP3 inflammasome activation.

### Manoalide Specifically Inhibits the NLRP3 Inflammasome Activation

We further explored whether manoalide impacts the LPS-priming process. 125-500 nM manoalide treatment before or after LPS stimulation did not impact the secretion of IL-6 and TNF-α induced by LPS (Supplementary Figures S2A,B). Also, the expression of NLRP3 and pro-IL-1β was not inhibited by manoalide treatment (Supplementary Figures S2C). Manoalide treatment did not affect the activation of NF-κB and MAPK induced by LPS, and the mRNA levels of IL-1β was not affected by manoalide treatment (Supplementary Figures S2D,E). These data indicated that manoalide inhibits the activation of NLRP3 inflammasome without affecting LPS-priming signaling.

**FIGURE 2:**
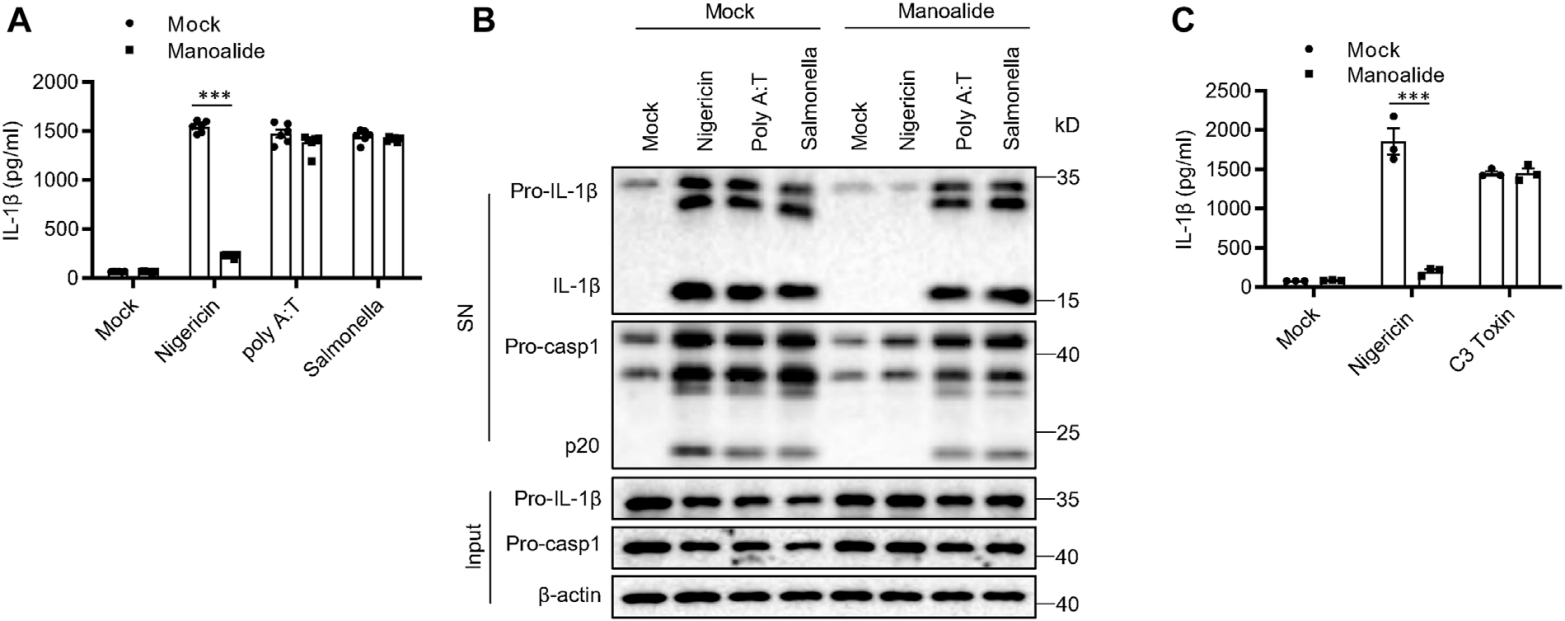
Manoalide has no effects on the activation of AIM2 inflammasome, IPAF inflammasome or Pyrin inflammasome. **(A)** ELISA of mature IL-1β and **(B)** Western blots of SN and Input from BMDMs primed with LPS and stimulated with nigericin, Poly (A:T) or *salmonella* (MOI = 10) with or without 500 nM manoalide. **(C)** ELISA of mature IL-1β in SN from BMDMs primed with LPS and then were pretreated with 500 nM manoalide before stimulation with C3 toxin. Data are derived from three independent experiments **(A,C)** and displayed as mean and SEM (*n* = 6) or represent three independent experiments **(B)**. Statistically significant difference was calculated by unpaired Student’s t-test: ^***^ *p* < 0.001.

There are other PRRs that can assemble inflammasomes except for NLRP3, including AIM2 inflammasome, IPAF inflammasome and Pyrin inflammasome (Mariathasan et al., 2004; Fernandes-Alnemri et al., 2009; Xu et al., 2014). Our data showed that poly (dA:dT)-induced AIM2 inflammasome activation and *S. typhimurium*-induced IPAF inflammasome activation are not affected by pretreatment with manoalide (Figures 2A,B). Manoalide did not inhibit Pyrin inflamasomes activated by C3 toxin (Figure 2C). Thus, manoalide is a highly specific NLRP3 inflammasome inhibitor.

### Manoalide Acts Downstream of Potassium Efflux, Chloride Efflux and Mitochondrial Dysfunction to Inhibit the Activation of NLRP3 Inflammasome Independently of PLA2/PLC Signaling

**FIGURE 3:**
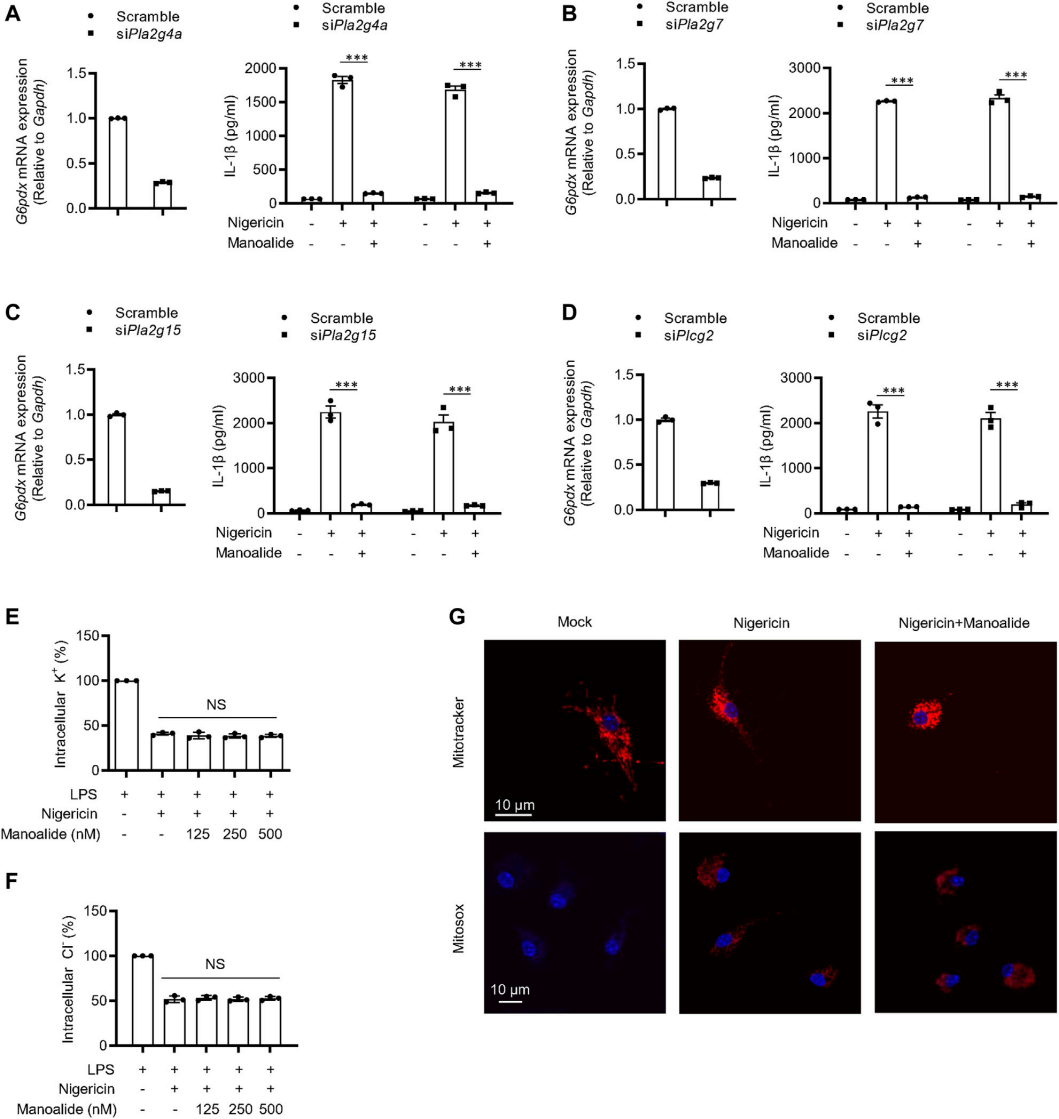
Manoalide has no effects on potassium efflux, chloride efflux or mitochondrial damage and inhibits the NLRP3 inflammasome activation independently of PLA2/PLC signaling. **(A–D)** PLA_2_ and PLC expression were knock down by siRNA in BMDMs. Then, the BMDMs were primed with LPS for 3 h and then were pretreated with 500 nM manoalide before stimulation with nigericin for 15 min **(A)** Pla2g4a, **(B)** Pla2g7, **(C)** Pla2g15 and **(D)** Plcg2 mRNA in BMDMs transfected with siRNA or control siRNA and ELISA of mature IL-1β in supernatant. Qualification of potassium efflux **(E)** and chloride efflux **(F)** in BMDMs primed with LPS and then were pretreated with 125, 250, 500 nM manoalide before stimulation with nigericin. **(G)** Confocal microscopy analysis of LPS-primed BMDM pretreated with 500 nM manoalide before stimulation with nigericin, then stained with MitoSOX red, Mitotracker red and DAPI. Data are derived from three independent experiments **(A–F)** and displayed as mean and SEM (*n* = 3) or represent three independent experiments **(G)**. Statistically significant difference was calculated by unpaired Student’s t-test: NS, not significant.

Then, we studied the potential mechanism of manoalide inhibiting the activation of NLRP3 inflammasome. Previous studies have shown that manoalide has analgesic and anti-inflammatory effects in a PLA2-dependent manner. And manoalide is also used as an inhibitor of PLC. Thus, we investigated whether the blockade of NLRP3 inflammasomes by manoalide depends on PLA2 or PLC. Among all the members in PLA2 and PLC family, PLA2G4A, PLA2G7, PLA2G15 and PLCG2 are highly expressed on BMDMs. We knocked down Pla2g4a, Pla2g7, Pla2g15 and Plcg2 with siRNA and found that the activation of NLRP3 inflammasome and the inhibitory effect of manoalide are not affected (Figures 3A–D). These results suggested that the inhibitory effect of NLRP3 inflammasome by maonoalide is independent of PLA2 or PLC.

It has been reported that potassium efflux and chloride efflux are critical upstream events in the activation of NLRP3 inflammsome (Muñoz-Planillo et al., 2013; Tang et al., 2017; Gong et al., 2018). Thus, we investigated whether the ion efflux is affected by manoalide. The concentration of intracelular potassium and chloride was significantly decline after nigericin stimulation. Manoalide did not affect nigericin or ATP-induced potassium and chloride efflux (Figures 3E,F, Supplementary Figures S3A,B). Previous studies have shown that mitochondrial damage and ROS production are additional upstream events in the activation of NLRP3 inflammasomes (Zhou et al., 2011; Gurung et al., 2015; Banoth and Cassel 2018; Elliott et al., 2018). By staining nigericin-stimulated BMDMs or human THP1 macrophage cell line, we observed that manoalide cannot reduce mitochondrial damage and ROS production, and (Figure 3G, Supplementary Figures S3C). Collectively, these results indicated that manoalide acts downstream of the ion flux, mitochondrial damage and ROS production to inhibit NLRP3 inflammasome activation.

### Manoalide Inhibits Assembly of the NLRP3 Inflammasome by Blocking the NEK7-NLRP3 Interaction

**FIGURE 4:**
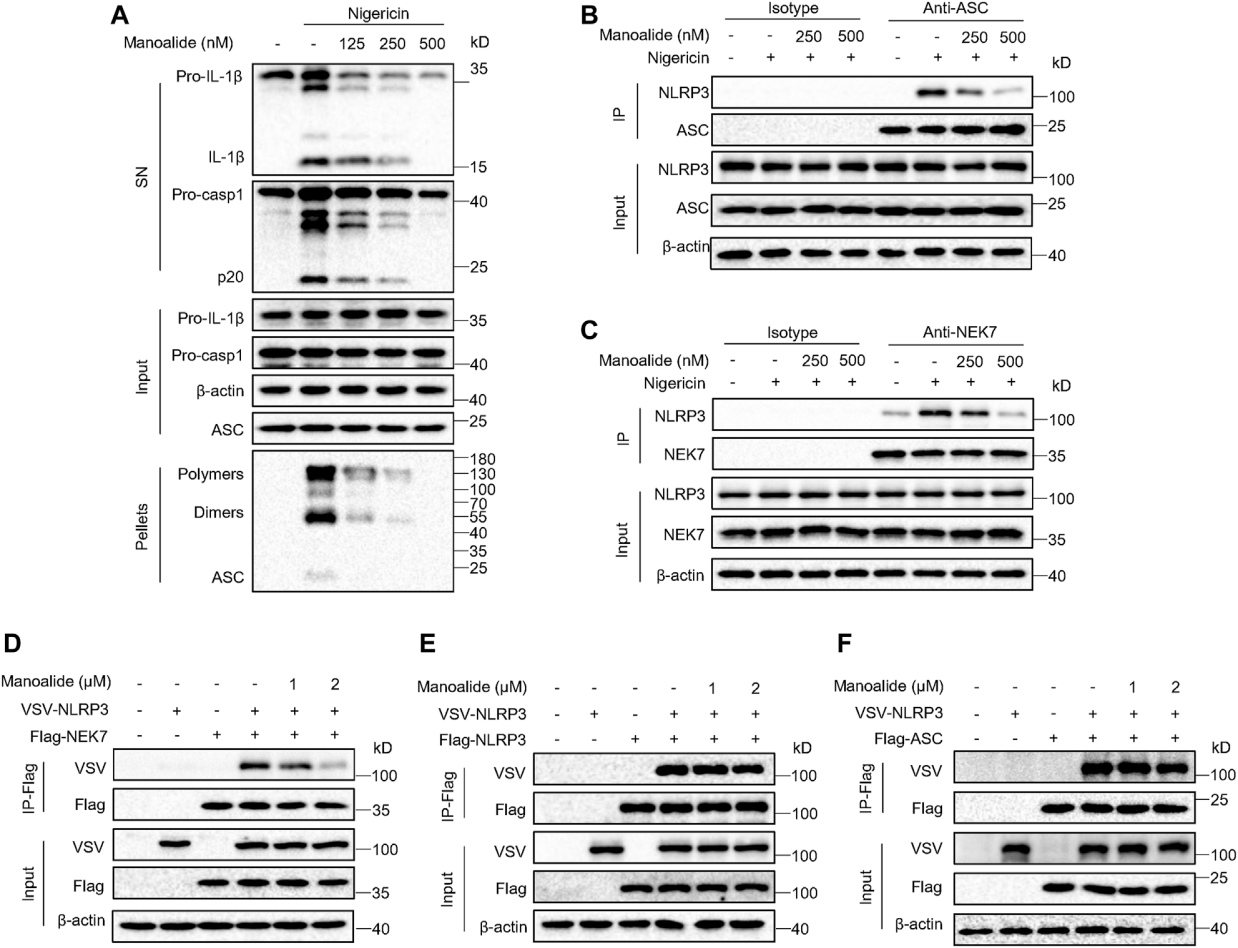
Manoalide blocks the assembly of NLRP3 inflammasome by inhibiting NLRP3-NEK7 interaction. **(A)** Western blot analysis of ASC oligomerization in the TBS buffer-insoluble pellets and lysates of LPS-primed BMDMs pretreated with 500 nM manoalide at different doses before stimulation with nigericin. **(B)** Western blot analysis of endogenous immunoprecipitation with ASC-antibody or isotype antibody in LPS-primed BMDMs pretreated with 250, 500 nM manoalide before stimulation with nigericin. **(C)** Western blot analysis of endogenous immunoprecipitation with NEK7-antibody or isotype antibody in LPS-primed BMDMs pretreated with 250, 500 nM manoalide before stimulation with nigericin. Western blot analysis of immunoprecipitation to evaluate **(D)** the NLRP3-NEK7 interaction, **(E)** the NLRP3-NLRP3 interaction and **(F)** the NLRP3-ASC interaction in the lysates of HEK-293T cells pretreated with 1, 2 μM manoalide. Data represent three independent experiments **(A-F)**.

To further investigate the mechanism of manoalide inhibiting the NLRP3 inflammasome activation, we supposed that manoalide might alters the assembly of the inflammasome complex. It has been reported that the ASC oligomerization is a key step in the effect phase and is essential for the subsequent caspase-1 cleavage (Lu et al., 2014; Dick et al., 2016). We found that nigericin-induced ASC oligomerization is dose-dependently inhibited by manoalide (Figure 4A), suggesting that manoalide targets the upstream of ASC oligomerization to block the maturation of caspase-1 and IL-1β. The NLRP3 oligomerization and recruitment of ASC are essential for the assembly of the NLRP3 inflammasome complex (Martinon et al., 2009; Davis et al., 2011). Through immunoprecipitation experiment, we found that nigericin induced a strong NLRP3-ASC interaction. Moreover, manoalide significantly attenuated the endogenous interaction between NLRP3-ASC (Figure 4B). NEK7 has been reported to interact with NLRP3, which is another important event for the oligomerization of NLRP3 and the recruitment of ASC (He et al., 2016). Our results showed that the endogenous NEK7-NLRP3 interaction is blocked in a dose-dependent manner by manoalide pretreatment (Figure 4C). Based on these results we considered that manoalide blocks the interaction between NEK7 and NLRP3, thereby inhibiting the assembly of NLRP3 inflammasome. To further verify this, we studied the interference of manoalide on the exogenous interaction of these proteins by the overexpression of FLAG-NEK7, VSV-NLRP3 and FLAG-ASC in HEK-293T cells. Indeed, we found that manoalide inhibits the NEK7-NLRP3 interaction in HEK-293T cells, but not the NLRP3-NLRP3 and NLRP3-ASC interaction (Figures 4D–F), suggesting that manoalide could not influence NLRP3 oligomerization and ASC recruitment. In addition, the ATPase activity of NLRP3 is critical for NLRP3 oligomerization (Duncan et al., 2007). Manoalide did not affect this event, either (Supplementary Figures S4A). Remarkably, previous research has shown NEK7-NEK9 interaction is essential for cell mitosis (Haq et al., 2015). Our results indicated that manoalide cannot weaken NEK7-NEK9 interaction (Supplementary Figures S4B). Consequently, these results demonstrated that manoalide prevents the assembly of NLRP3 inflammasome by diametrically restraining the NEK7-NLRP3 interaction.

### Manoalide Interacts With NLRP3 via Covalent Binding

**FIGURE 5:**
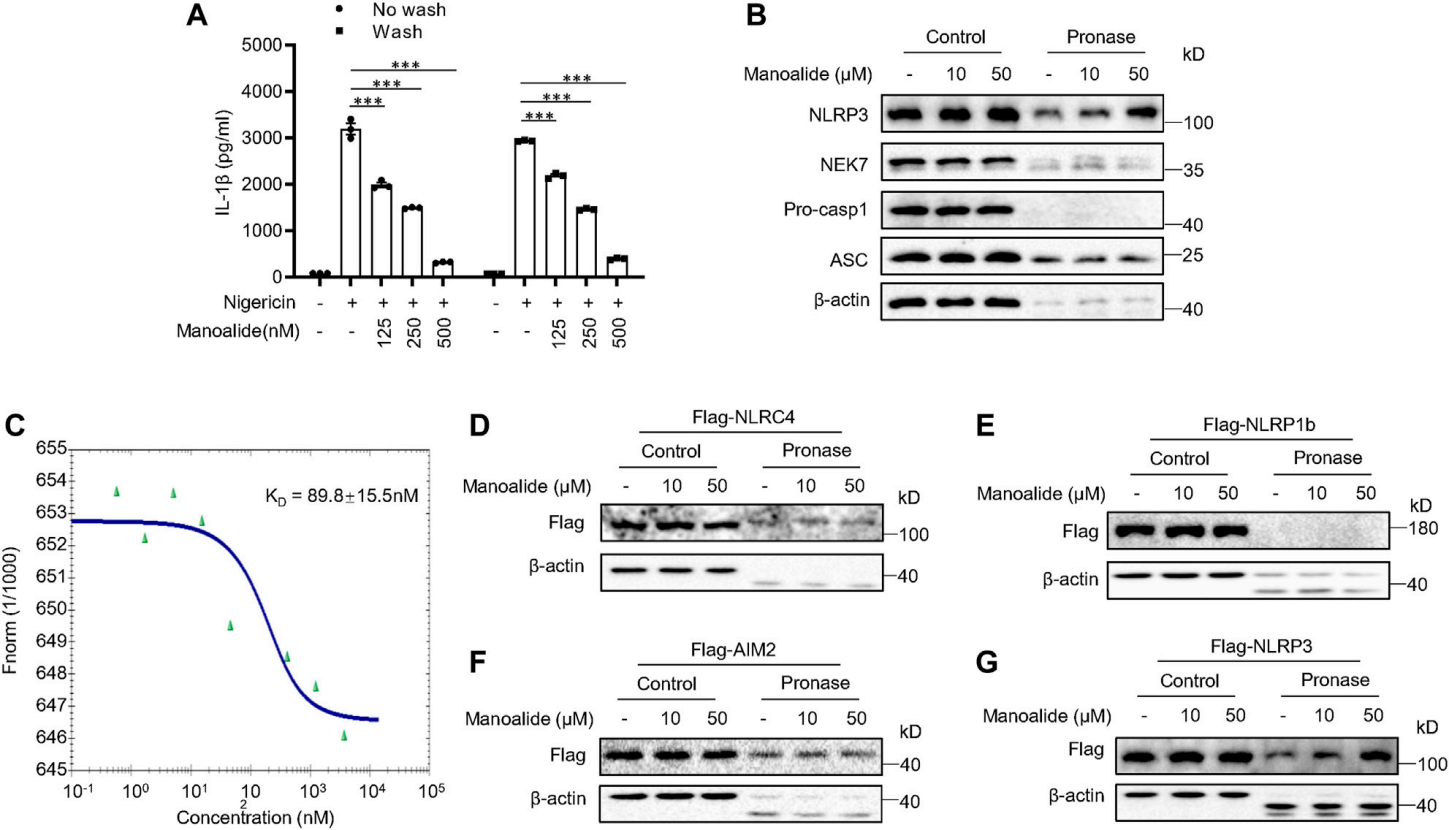
Manoalide irreversibly binds to NLRP3. **(A)** ELISA of mature IL-1β in the supernatant of LPS-primed BMDMs pretreated with 125, 250, 500 nM manoalide and then were washed three times before stimulation with nigericin. **(B)** DARTS analysis of NLRP3, NEK7, pro-caspase-1 and ASC in lysates from LPS-primed BMDMs incubated with 10, 50 μM manoalide. **(C)** MST assay was performed to evaluate the affinity between manoalide with purified His-GFP-NLRP3. DARTS analysis of **(D)** Flag-NLRC4, **(E)** Flag-NLRP1b, **(F)** Flag-AIM2, **(G)** Flag-NLRP3 from lysates of HEK-293T cells transfected with indicated plasmids in the presence or absence of 10, 50 μM manoalide. Data are derived from three independent experiments **(A)** and displayed as mean and SEM (*n* = 3) or represent three independent experiments **(B-G)**. Statistically significant difference was calculated by unpaired Student’s t-test: ^***^ *p* < 0.001.

Next, we searched for the target protein of manoalide. Since we have verified that manoalide inhibits the assembly of NLRP3 inflammasome complex by preventing NEK7-NLRP3 interaction, we suspected that NEK7 or NLRP3 is the target protein of manoalide. First, we tested whether the inhibitory effect of manoalide on NLRP3 inflammasomes is reversible. Before stimulating, we pretreated LPS-primed BMDMs with manoalide for 15 min, then washed the cells to remove unbound molecules. The elution did not affect the suppression of manoalide on nigericin-induced IL-1β secretion (Figure 5A), indicating that the inhibition of NLRP3 inflammasome activation by manoalide pretreatment is irreversible. The drug affinity responsive target stability (DARTS) is based on the characteristic that the protein’s sensitivity to proteases is reduced after the drug binds to the target protein (Lomenick et al., 2009). Therefore, we identified the target protein that binds to manoalide by DARTS, using BMDMs and pronase. Pronase-mediated degradation of NLRP3 is reversed in dose-dependent manner by manoalide. However, the degradation of other components of the NLRP3 inflammasome such as NEK7, ASC and caspase-1 was not affected (Figure 5B). These results suggested manoalide specifically binds to NLRP3. We also overexpressed and purified HIS-GFP-NLRP3 protein in HEK-293T cells. Microscale thermophoresis (MST) assay showed that the equilibrium dissociation constant (K_D_) between manoalide and purified HIS-GFP-NLRP3 was ∼89.8 nM (Figure 5C), indicating manoalide directly binds to NLRP3 with high affinity.

Furthermore, we investigated whether manoalide interacts with other PRRs that can form the inflammasome complex. We expressed NLRP3 and other inflammasome sensors in HEK-293T. The DARTS assay revealed that manoalide specifically interacts with NLRP3, but not AIM2, NLRC4 and NLRP1b (Figures 5D–G). Consequently, these results suggested that manoalide irreversibly and selectively binds to NLRP3.

### Manoalide Targets Lysine 377 of NLRP3 NACHT Domain

**FIGURE 6:**
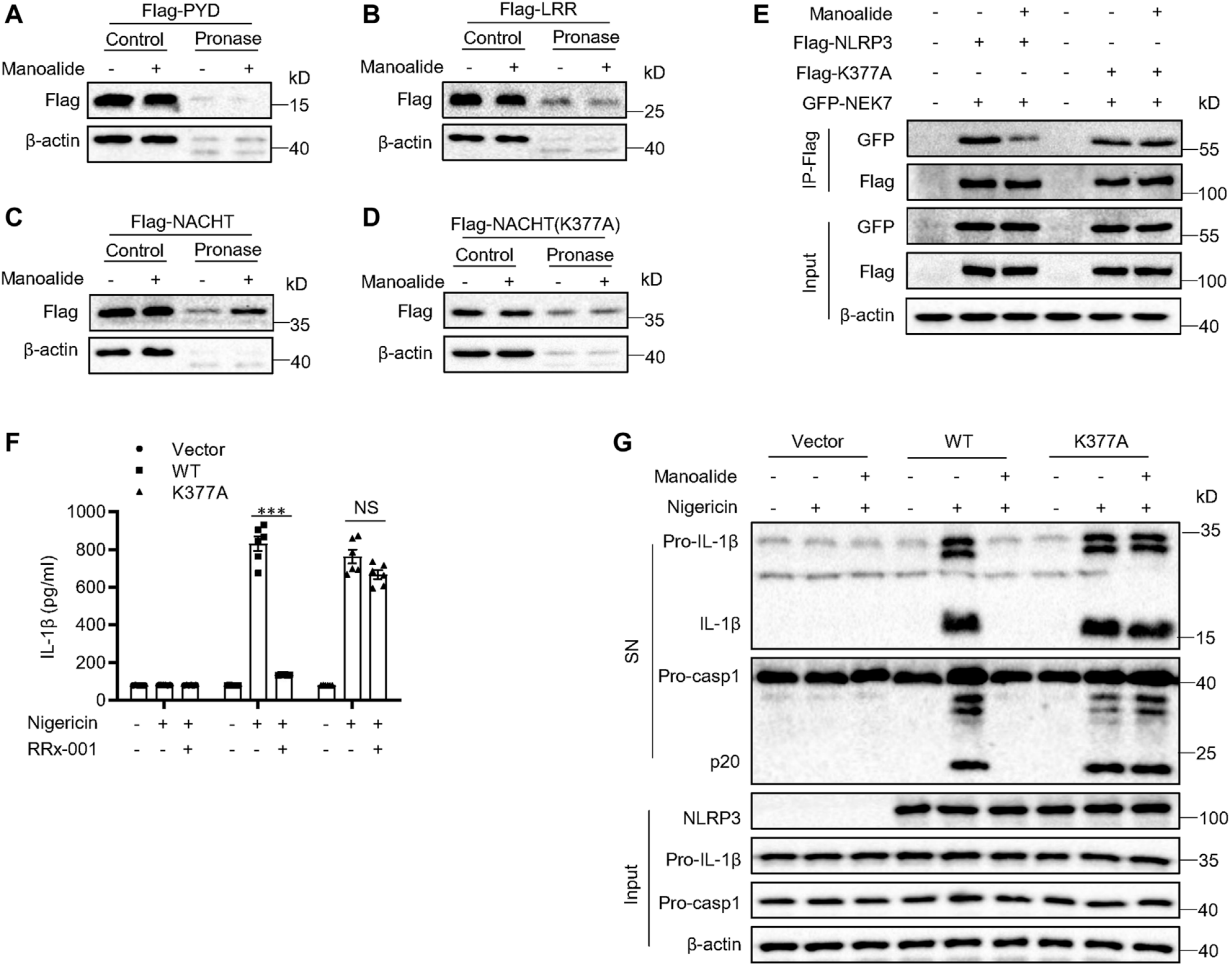
Manoalide binds to lysine 377 of NLRP3. DARTS analysis of **(A)** Flag-NACHT, **(B)** Flag-PYD, **(F)** Flag-LRR, **(G)** Flag-NACHT (C409A) from lysates of HEK-293T cells transfected with indicated plasmids in the presence or absence of 50 μM manoalide. **(E)** Western blot analysis of immunoprecipitation to evaluate the NLRP3-NEK7 interaction or NLRP3(K377A)-NEK7 interaction in the lysates of HEK-293T cells pretreated with 2 μM manoalide. **(F)** ELISA of mature IL-1β or **(G)** western blot analysis of the mature IL-1β and activated caspase-1 in supernatant from LPS-primed Nlrp3^−/−^ BMDMs reconstituted with WT NLRP3 or NLRP3 K377A that were pretreated with 500 nM manoalide before stimulation with nigeicin. Data are derived from three independent experiments **(F)** and displayed as mean and SEM (*n* = 3) or represent three independent experiments **(A-E,G)**. Statistically significant difference was calculated by unpaired Student’s t-test: ^***^ *p* < 0.001; NS, not significant.

Next, we investigated the binding site of manoalide on NLRP3. NLRP3 is a tripartite structure, consisting of the C-terminal LRRs-domain, the central NACHT domain and the N-terminal PYD-domain (Lechtenberg et al., 2014). We constructed the three separate domain plasmids and expressed them in HEK-293T cells. Subsequent DARTS experiments displayed that manoalide binds to the NACHT domain of NLRP3, but not to the LRRs domain or PYD domain (Figures 6A–C).

It has been described that manoalide binds permanently to lysine residues. Thus, we hypothesized that manoalide may bind to lysine residues in the NACHT domain. The NACHT domain of NLRP3 contains 18 lysines according to BLAST analysis. Most of these lysine sites are conserved in evolution. Considering that there are many lysine sites in the NACHT domain, we constructed two NACHT domain truncations and expressed them in HEK-293T cells in order to narrow the scope (Supplementary Figures S5A). Manoalide could bind to both NACHT domain truncations (Supplementary Figures S5B), suggesting that manoalide targets HD1 region of the NACHT domain. Combining with the result of the previous BLAST sequence analysis, we found six lysine residues. In order to confirm which lysine residue binds to manoalide, we constructed NACHT mutants, in which a lysine was replaced with an alanine. The DARTS results displayed that only the mutant at lysine 377 (K377A) reversed NACHT binding to manoalide (Figure 6D), and all other mutants were interacted with manoalide (Supplementary Figures S5C).

To confirm that manoalide binds to the lysine 377 sit of NLRP3 to block the NLRP3 inflammsome activation, we constructed the FLAG-NLRP3 K377A mutant plasmid. Exogenous co-immunoprecipitation experiment results showed that the K377A mutation did not affect the NEK7-NLRP3 interaction, but it abolished the inhibition of NEK7-NLRP3 interaction by manoalide pretreatment (Figure 6E). We expressed WT NLRP3 or NLRP3 (K377A) in Nlrp3^−/−^ BMDMs. Both WT NLRP3 and NLRP3 (K377A) could promote the cleavage of caspase-1 and secretion mature IL-1β. Consistently with the previous results, manoalide could not inhibit the activation of NLRP3 inflammasome reconstructed by NLRP3(K377A) (Figures 6F,G). Collectively, these results clearly demonstrated that the lysine 377 of NLRP3 is the binding site of manoalide.

### Manoalide Attenuates Mouse Experimental Autoimmune Encephalomyelitis Models by Suppressing NLRP3-Driven Inflammation *in vivo*

**FIGURE 7:**
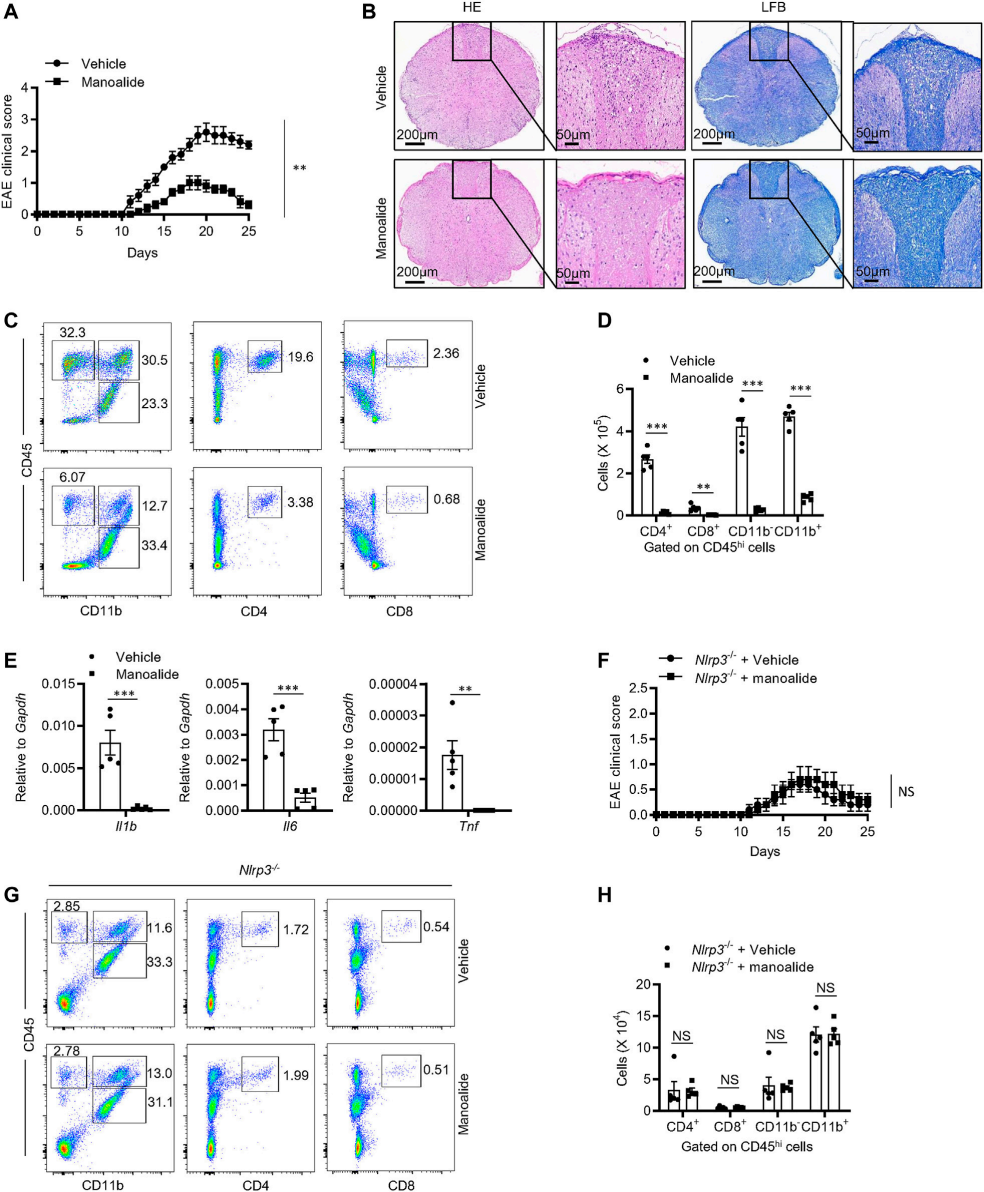
Manoalide attenuates the severity of EAE in an NLRP3-dependent manner. **(A-E)** WT mice were injected intraperitoneally with manoalide (5 mg/kg) or PBS every 3 days after induction of EAE. **(A)** Clinical scores was assessed after EAE induction. **(B)** Sections of paraffin-embedded spinal cord tissues from mice in the presence or absence of manoalide after EAE induction were stained with H&E or Luxol fast blue (LFB). **(C)** FACS analysis of live CD4^+^, CD8^+^, CD11b^+^ and CD11b^−^ cells gate on CD45^hi^ cells in the CNS on the 15 days after induction of EAE. **(D)** The numbers of live CD4^+^, CD8^+^, CD11b^+^ and CD11b^−^ cells gate on CD45^hi^ cells in the CNS on the 15 days after induction of EAE. **(E)** The mRNA levels of IL-1β, IL-6 and TNF-α in CNS were evaluated by real-time PCR. **(F-H)** Nlrp3−/− mice were injected intraperitoneally with manoalide (5 mg/kg) or PBS every 3 days after induction of EAE. **(F)** Clinical scores was assessed after EAE induction. **(G)** FACS analysis of live CD4^+^, CD8^+^, CD11b^+^ and CD11b^−^ cells gate on CD45^hi^ cells in the CNS on the 15 days after induction of EAE. **(H)** The numbers of live CD4^+^, CD8^+^, CD11b^+^ and CD11b^−^ cells gate on CD45^hi^ cells in the CNS on the 15 days after. Data are displayed as mean and SEM (*n* = 5) **(A,D-F,H)** or represent 5 mice **(B,C,G)**. Statistically significant difference was calculated by unpaired Student’s t-test: ^**^ *p* < 0.01; ^***^ *p* < 0.001; NS, not significant.

Given manoalide strongly inhibited the activation of NLRP3 inflammasome *in vitro*, we then investigated the inhibitory effects of manoalide *in vivo*. Experimental allergic encephalomyelitis (EAE), characterized by CD4^+^ T cells-mediated inflammation and demyelination, is an ideal animal model for human multiple sclerosis (MS) (Constantinescu et al., 2011). Previous studies reported that the IL-1 signaling is essential for the production of pathogenic inflammatory factor IL-17 from Th17 and γδT cells (Gris et al., 2010). Recently, more and more evidences proved that NLRP3 is critical to the development of EAE. Thus, we considered whether manoalide ameliorates the progression of EAE by inhibiting NLRP3 inflammasomes activation. Mice had been treated with manoalide or PBS every 3 days by intraperitoneal injection since the EAE model was induced. The results showed that manoalide treatment postpons the onset time and decreases the condition serious degree of EAE in mice (Figure 7A). By staining mice spinal cord with H&E or Luxol fast blue (LFB), we found that treatment with manoalide decreases the infiltration of inflammatory cells and demyelination in the spinal cord compared to the control group (Figure 7B). In addition, at the peak of the onset time of EAE, we isolated the immune cells infiltrating in the central nervous system and employed flow cytometric analysis. FACS analysis showed that the abundances and numbers of CD11b^+^, CD11b^−^, CD4^+^ and CD8^+^ cells infiltrated in the CNS of mice treated with manoalide were significantly lower than those of control group (Figures 7C,D and Supplementary Figures S6A). We also tested the expression of proinflammatory cytokines, including IL-1β, IL-6 and TNF-α, in the spinal cord by quantitative real-time PCR. As expected, manoalide treatment dramatically reduced the expression of these proinflammatory factors (Figure 7E). These results suggested that manoalide alleviates the CNS inflammation and severity of the disease, thereby protects mice from EAE.

In order to verify that the therapeutic effect of manoalide on EAE depends on NLRP3, we induced EAE on Nlrp3^−/−^ mice and treated them with manoalide or PBS. Consistently with previous reports, Nlrp3^−/−^ mice were resistant to EAE, including the decrease in clinical score and the numbers of infiltrating immune cells in CNS. Treatment with manoalide could not further attenuate the pathogenesis and the severity of EAE in Nlrp3^−/−^ mice (Figures 7F–H), indicating that the therapeutic effect of manoalide in EAE depends on its inhibition of the NLRP3 inflammasome activation.

## Discussion

In this study, we demonstrated that manoalide can effectively inhibit the activation of NLRP3 inflammasome and ameliorate the pathogenesis of experimental autoimmune encephalomyelitis in mice. This finding showed that manoalide has the potential to be a new therapeutic approach for NLRP3-related diseases. Besides, we found an alternative mechanism of manoalide inhibiting inflammation through NLRP3.

Our results indicated that manoalide directly binds to the lysine 377 site of NLRP3 and inhibit the assembly of the NLRP3 inflammasome. Manoalide specifically inhibited the NLRP3 inflammasome activation downstream of potassium efflux, chloride efflux and mitochondrial dysfunction in a PLA2/PLC-independent manner. Collectively, manoalide is a direct inhibitor of NLRP3. Several inhibitors targeting NLRP3 have been reported recently, but there is no drug clinically available for treatment of NLRP3-driven diseases. We found that manoalide efficiently inhibits the NLRP3 inflammasome activation *in vitro* and *in vivo*. In mouse macrophages and human monocytes, treatment with 500 nM of manoalide could totally abrogate the NLRP3 inflammasome activation. In mouse EAE model, intraperitoneal administration of manoalide at a dosage of 5 mg/kg every 3 days was able to significantly attenuate the neuroinflammation and severity of the disease. Manoalide was once approved for clinical trials for the treatment of psoriasis which was suspended in Phase II clinical trial due to formulation issues, indicating that it could be a safe agent with high efficiency and low toxicity (Kijjoa and Sawangwong 2004).

It has been reported that manoalide inhibits the inflammation by targeting PLA2 with an IC50 value of 60 μM. Our results indicated that 100–500 nM of manoalide could obviously inhibit the secretion of pro-inflammatory cytokine IL-1β induced by nigericin stimulation. Furthermore, manoalide inhibited the inflammation by covalently binding to NLRP3. Therefore, we found a new mechanism of manoalide inhibiting inflammatory response and a new target of manoalide.

Manoalide has been demonstrated to be a calcium channel blocker. The mobilization of Ca^2+^ induced by NLRP3 agonists increases the concentration of cytosolic Ca^2+^, leading to mitochondrial damage and the inflammasome activation (Horng 2014). However, our data showed that manoalide inhibits the NLRP3 inflammasome activation without affecting the mitochondria, indicating that manoalide does not affect the calcium flow during this process.

Manoalide contains three functional groups, including γ-hydroxybutenolide, α-hydroxydihydropyran and the trimethylcyclohexenyl. The α-hydroxydihydropyran is necessary for the irreversible binding of manoalide to lysines of PLA2, and the hydrophobic trimethylcyclohexenyl ring further enhances the interaction. We found that manoalide binds to NLRP3, but the functional group for the interaction remains unknown. Our data showed that the binding site of manoalide on NLRP3 (Lys 377) is necessary for the interaction between NLRP3 and NEK7. We hypothesized that manoalide may directly target the NEK7-NLRP3 interaction interface. However, according to the structure of NEK7-NLRP3 interaction analyzed by cryo-electron microscope (Sharif et al., 2019), lysine 377 is not on the NEK7-NLRP3 binding interface. Thus, the structural mechanism of manoalide inhibiting the activation of NLRP3 inflammasome needs to be further investigated.

Covalent drugs have stronger efficacy and longer duration of action. We found that manoalide is a covalent binding inhibitor of NLRP3 and has therapeutic effects on EAE in mice. Thus, manoalide and its analogs have great potential to treat NLRP3-associated diseases, especially the CNS diseases.

## Data Availability

The original contributions presented in the study are included in the article/Supplementary Material, further inquiries can be directed to the corresponding authors.
