# Supplementary material for: Inhibition of the NLRP3 Inflammasome Activation by Manoalide Ameliorates Experimental Autoimmune Encephalomyelitis Pathogenesis
Source: Front Cell Dev Biol. 2022 Feb 16;10:822236. doi: 10.3389/fcell.2022.822236 (PMC8888861; doi:10.3389/fcell.2022.822236)
Supplement: Supplementary file 1 [file DataSheet1.PDF]

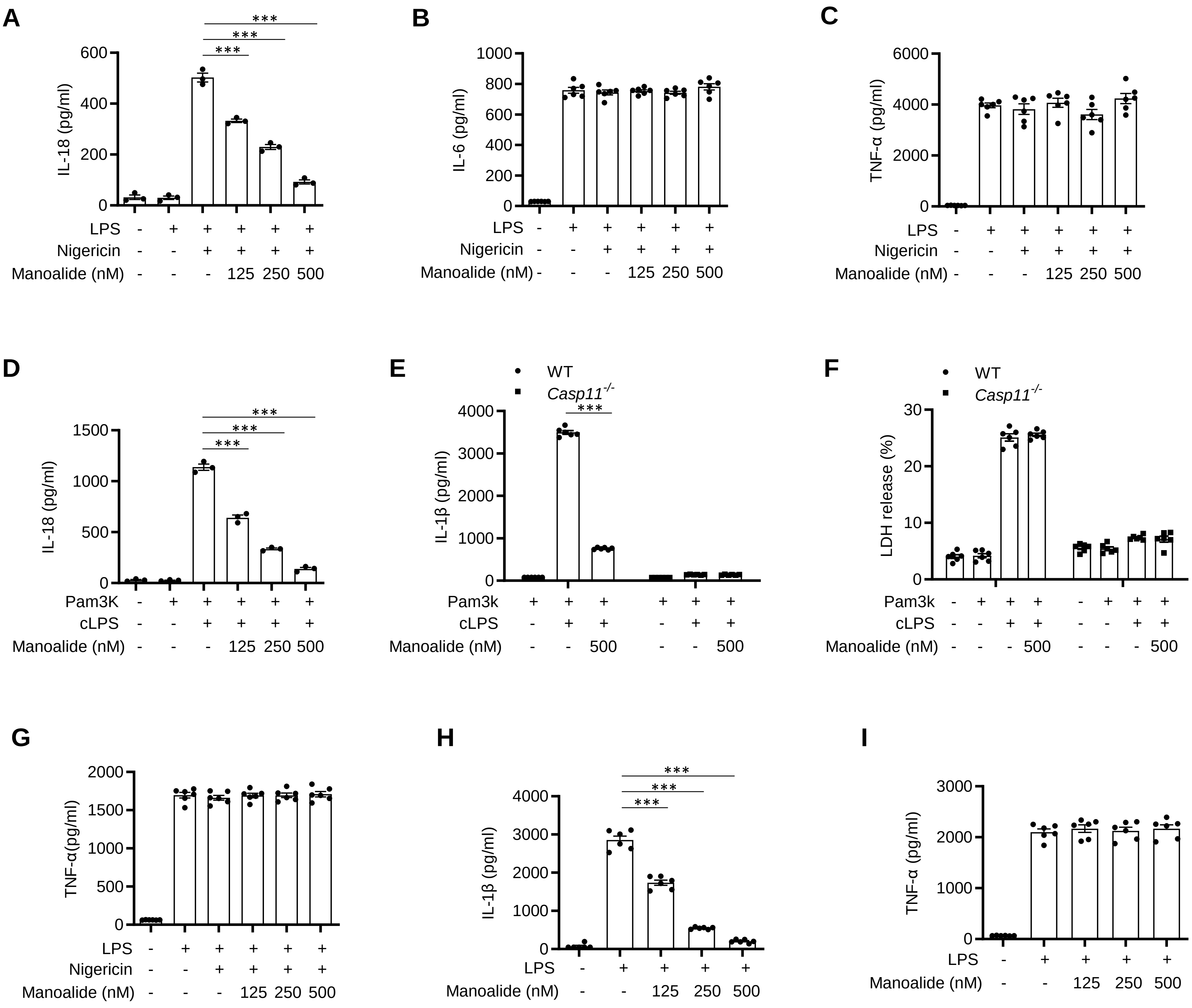

Supplementary Figure 1

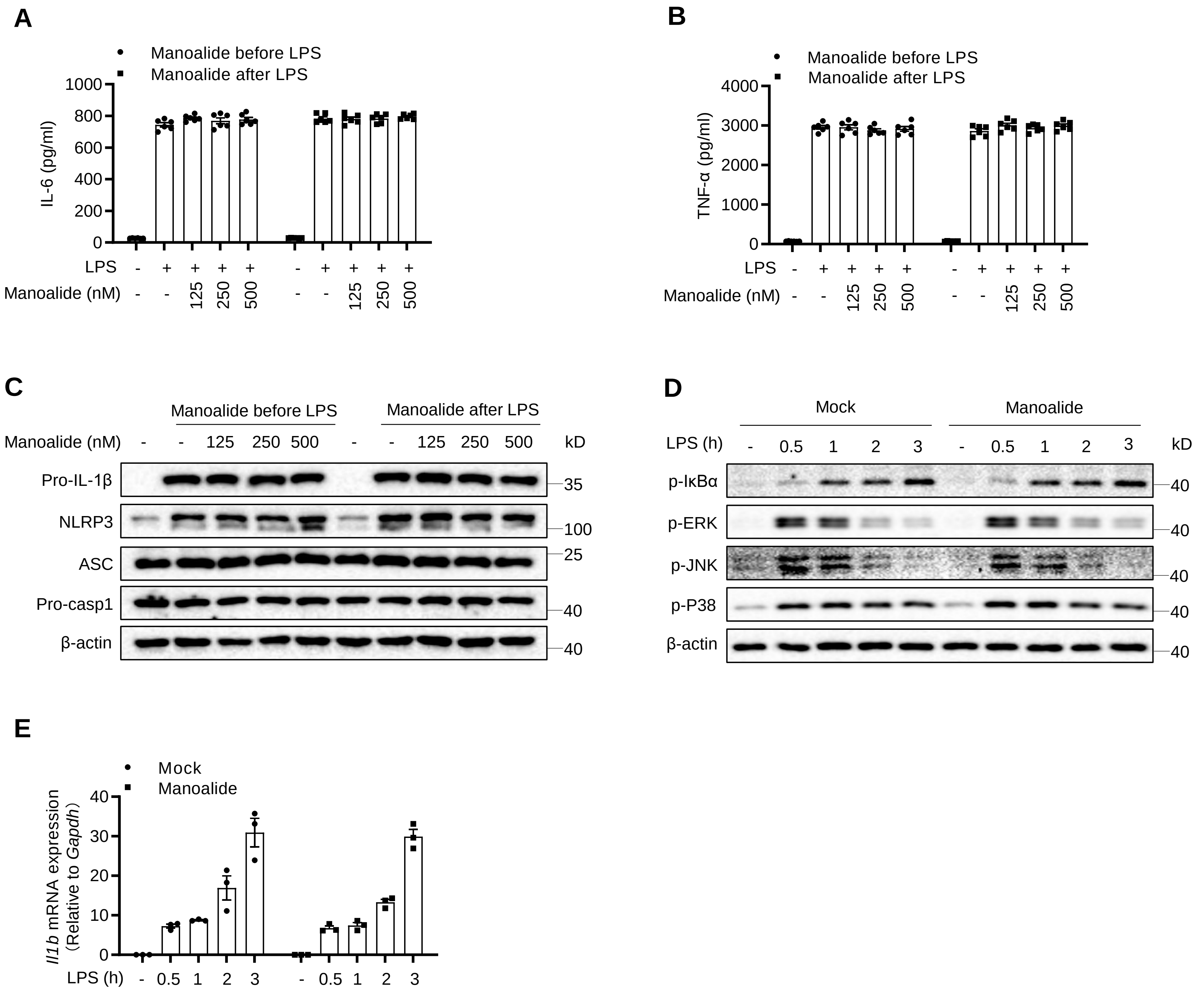

Supplementary Figure 2

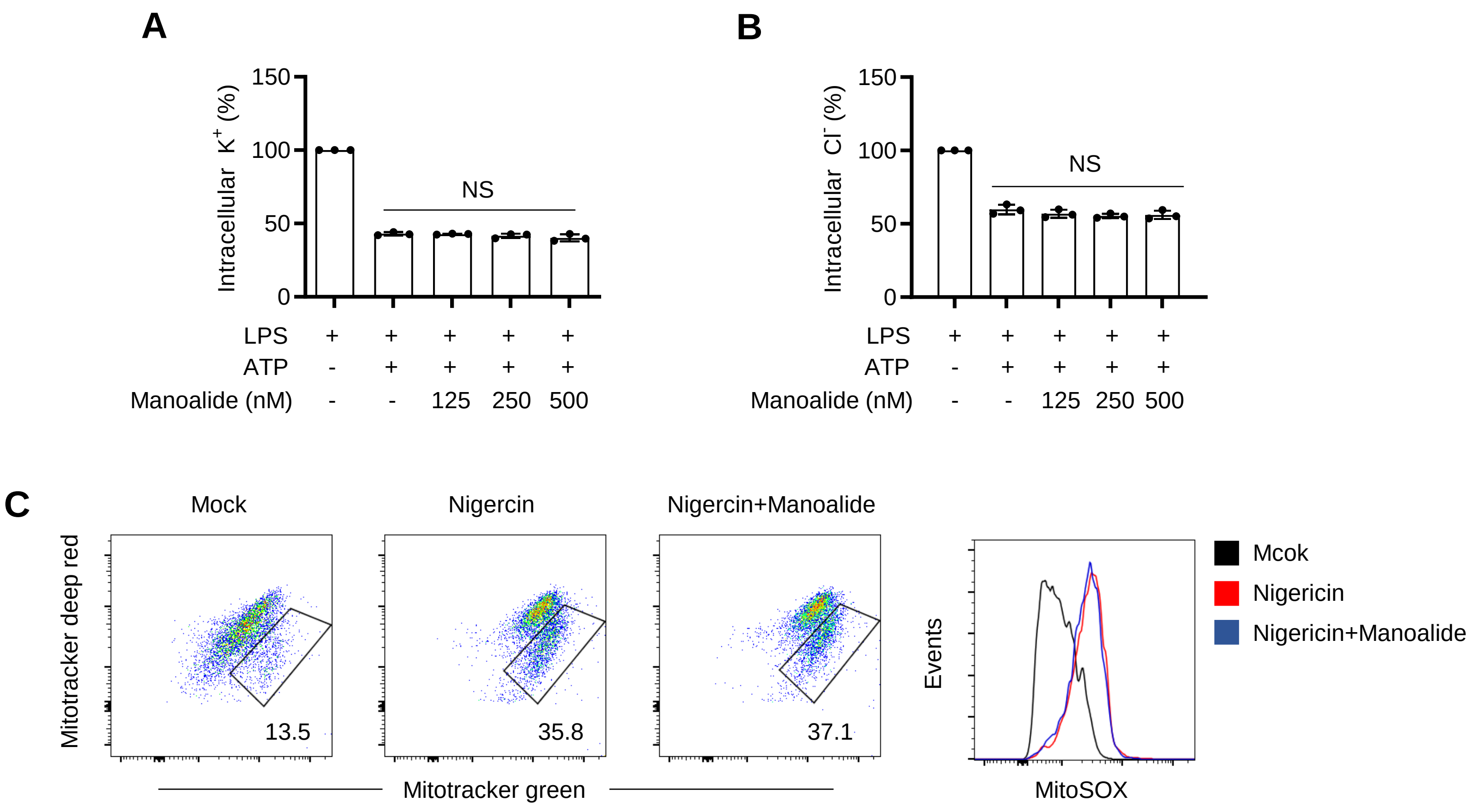

Supplementary Figure 3

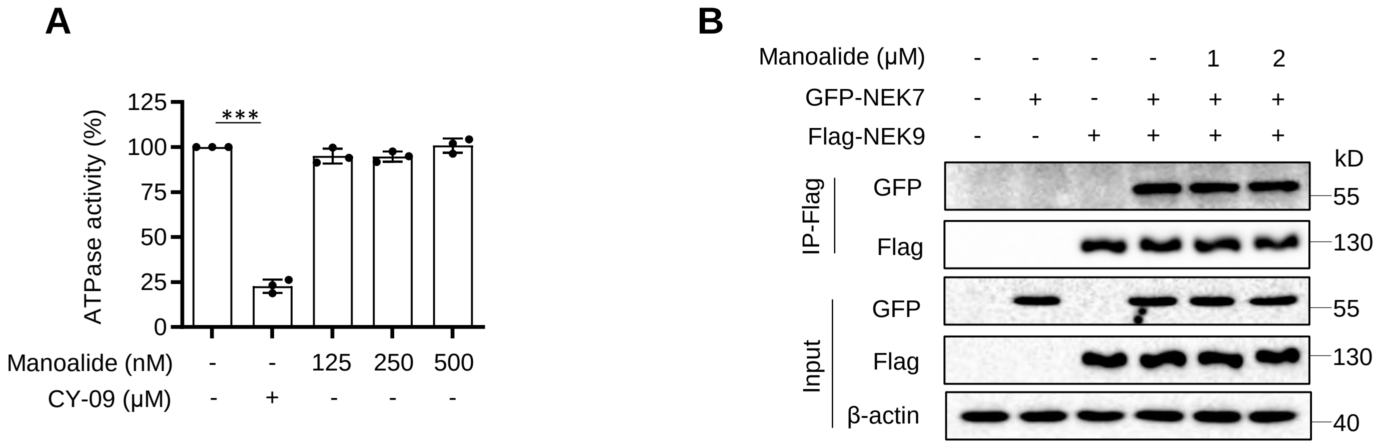

Supplementary Figure 4

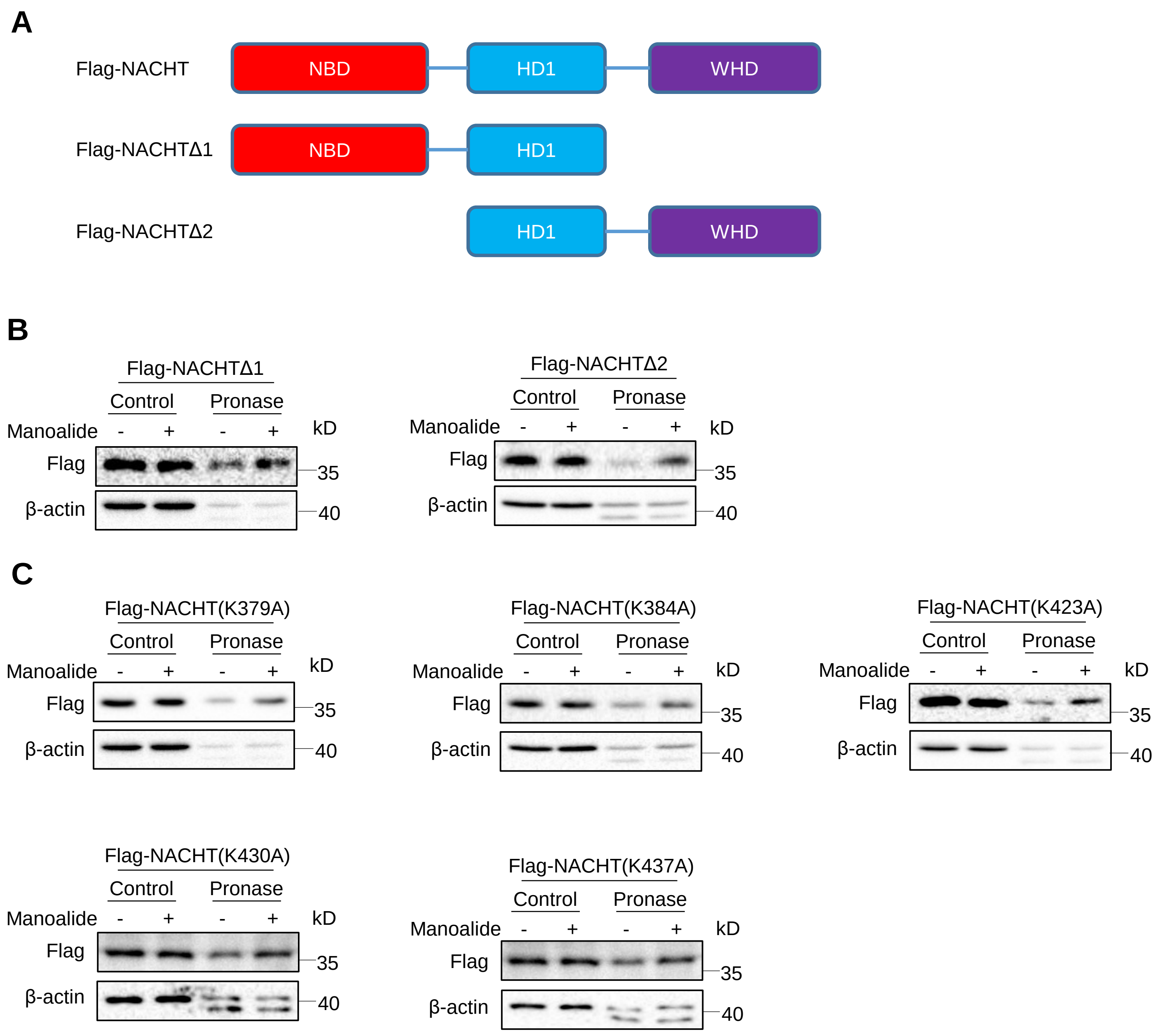

Supplementary Figure 5

A

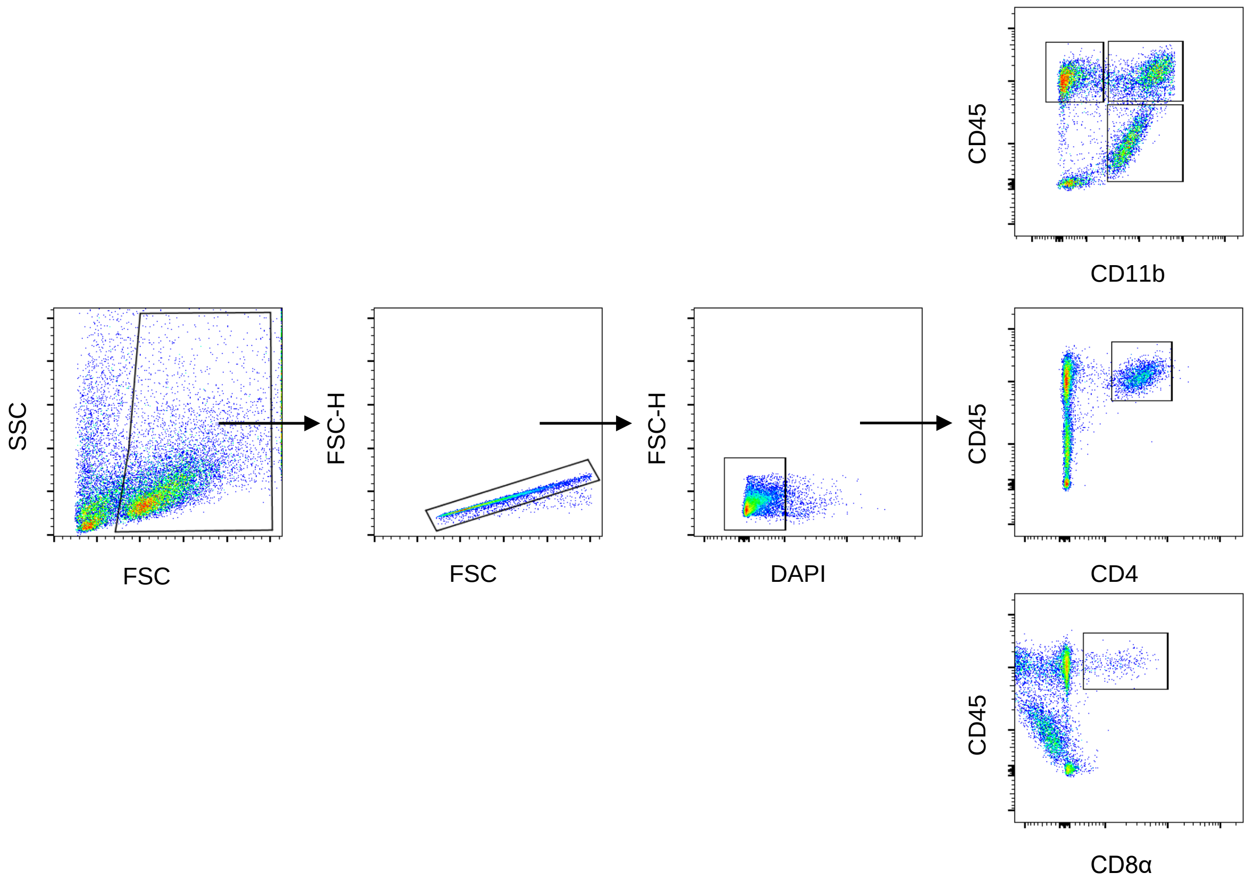

Supplementary Figure 6
